# In Silico Prospecting for Novel Bioactive Peptides from Seafoods: A Case Study on Pacific Oyster (*Crassostrea gigas*)

**DOI:** 10.3390/molecules28020651

**Published:** 2023-01-09

**Authors:** Leyi Zhou, Rufa L. Mendez, Jung Yeon Kwon

**Affiliations:** 1 Department of Food Science and Technology, College of Agricultural Sciences, Oregon State University, Corvallis, OR 97333, USA; 2 Seafood Research and Education Center, Oregon State University, Astoria, OR 97103, USA

**Keywords:** in silico prospecting, bioactive peptides, seafood, *Crassostrea gigas*, oyster proteins

## Abstract

Pacific oyster (*Crassostrea gigas*), an abundant bivalve consumed across the Pacific, is known to possess a wide range of bioactivities. While there has been some work on its bioactive hydrolysates, the discovery of bioactive peptides (BAPs) remains limited due to the resource-intensive nature of the existing discovery pipeline. To overcome this constraint, in silico-based prospecting is employed to accelerate BAP discovery. Major oyster proteins were digested virtually under a simulated gastrointestinal condition to generate virtual peptide products that were screened against existing databases for peptide bioactivities, toxicity, bitterness, stability in the intestine and in the blood, and novelty. Five peptide candidates were shortlisted showing antidiabetic, anti-inflammatory, antihypertensive, antimicrobial, and anticancer potential. By employing this approach, oyster BAPs were identified at a faster rate, with a wider applicability reach. With the growing market for peptide-based nutraceuticals, this provides an efficient workflow for candidate scouting and end-use investigation for targeted functional product preparation.

## 1. Introduction

Bioactive peptides (BAPs) are short peptides, usually 2–20 residues in length [1], that can exhibit a positive impact on human health through mitigating various molecular pathways implicated in chronic disease development. Dietary proteins are a widely available and accessible source of bioactive peptides. The human digestive system can, among many other methods such as food processing, fermentation, enzymatic and chemical hydrolysis [2], effectively release cryptic BAPs within food proteins through the hydrolytic action by digestive proteases. Therefore, the bioactive potential of plant and animal proteins liberated during digestion has been widely studied through in vitro, in vivo, and in silico approaches [3,4]. Many functional food products and ingredients that carry food-derived bioactive peptides are commercially available [5].

Marine organisms have gained tremendous research interest in their bioactivity and therapeutic potential [6,7]. Oysters are the most cultured shellfish worldwide [8] and are traditionally used as medicine for various purposes in Asia [9]. They are nutrient-dense, high in protein, low in fat, and rich in various micronutrients and bioactive components. Pacific oyster (*Crassostrea gigas*) has shown promise experimentally exhibiting various bioactivities including antimicrobial, antioxidant, anti-inflammatory, angiotensin-converting enzyme (ACE)-inhibitory, anti-cancer, and promotion of sexual reproductive hormones [8,10,11]. Therefore, *C. gigas* demonstrates immense opportunity as a BAP precursor in a wide range of bioactivity classes for functional food and nutraceutical application. BAPs bring key advantages including high target specificity, potency, and low toxicity compared to small molecule drugs. However, the main challenges in translating BAP research remain their questionable bioavailability, stability in digestion and circulation, and bitterness [12,13]. To circumvent these disadvantages in new BAP discovery, in silico screening and prediction methods may be applied. Many studies examining bioactive peptides have effectively incorporated in silico methods to accelerate the conventional BAP discovery process, thereby achieving higher precision, time, cost, and resource savings [14,15,16,17]. While there are many studies mining the bioactive potential and BAP sequences from *C. gigas* and related species, few have integrated in silico methods to cover a wider candidate pool than the physical samples and available reagents may allow [15,18]. In this present study, we set out to identify novel BAP candidates from *C. gigas* flesh released by human consumption using in-silico screening methods and develop a transferrable pipeline for the in silico discovery of BAPs from GI-digested food proteins.

## 2. Results

### 2.1. Simulated Gastrointestinal Digestion 

**Figure 1:**
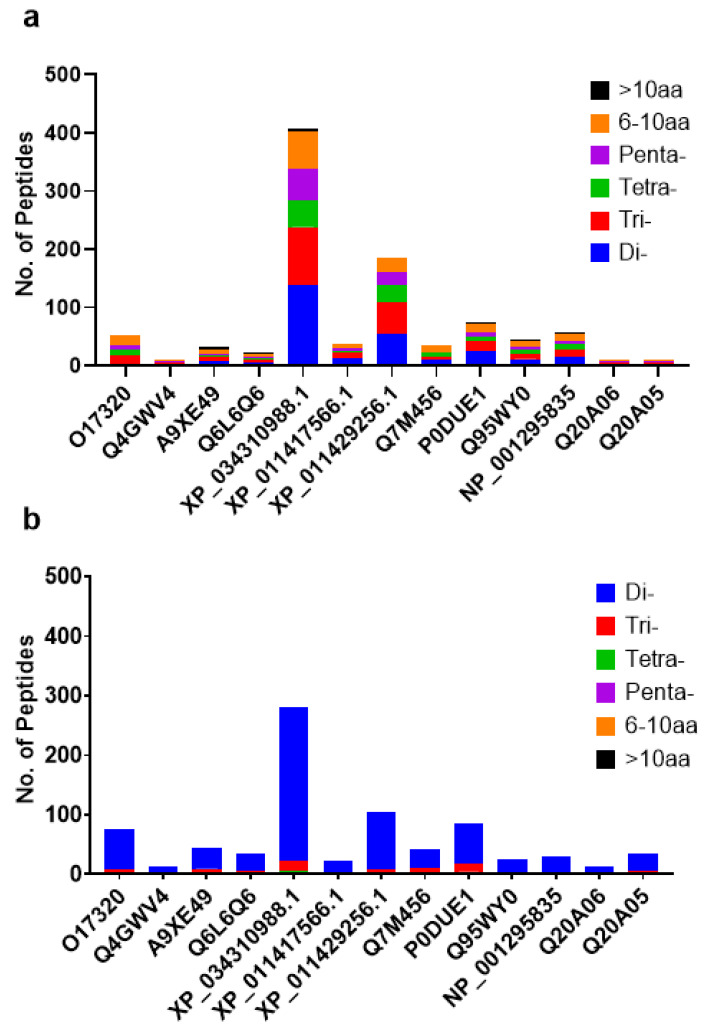
Virtual peptide products from 13 digested oyster proteins using (**a**) ExPASy PeptideCutter and (**b**) BIOPEP-UVM’s “Enzyme Action” tool.

The enzymatic hydrolysis on all 13 selected *C. gigas* proteins with human digestive enzymes, pepsin, trypsin, and chymotrypsin (Table 1) resulted in 1800 total protein fragments (excluding single amino acids) via BIOPEP Enzyme Action Tool and ExPASy PeptideCutter combined. The peptide lengths ranged from 2 to 16 residues. The resulting peptide profiles from each protein are shown in Figure 1. To maximize the potential for novelty and specificity to oyster origin while maintaining bioavailability, only peptides of 5 to 10 residues in length were selected for further analyses, which narrowed down the candidate pool to a total of 289 unique peptide sequences. ToxinPred and iBitter-SCM revealed 126 bitter peptides, one of which is also predicted to be toxic. Only the remaining 163 unique non-toxic, non-bitter peptides were considered for further analysis. HLP analysis shows that all but two remaining peptide candidates have high intestinal stability, defined by half-life > 1.0 s, while PLifePred analysis predicted plasma half-life ranging from 757.01 to 1058.51 s. Most natural food-derived peptides have a half-life between 800 to 900 s based on PLifePred from Gülseren and Vahapoglu’s [19] investigation of 3074 peptides from 12 different food sources. Therefore, a threshold half-life of 800 s or above was chosen to benchmark for relatively high *C. gigas* peptide stability in the blood. From the multiple database scans, only one sequence AGDDAPR from the peptide list was previously reported as a bioactive peptide record in BIOPEP and EROP-Moscow databases, with ACE-inhibiting, antioxidative, Pancreatic lipase-inhibiting, and alpha-amylase-inhibiting activities. With our comprehensive screening phase detailed above, 151 nontoxic, non-bitter, highly stable, and novel sequences remained for bioactivity screening in silico, shown in Table 2.

### 2.2. Prospect Oyster BAPs

From the BAP candidate pool (151 sequences), 22 were shortlisted based on their predicted bioactivities in various in silico platforms performed (Table 3). Sequences were considered high potential/prospect BAPs when they gain scores beyond the established predictive model threshold and when these consistently show interaction on some known binding sites associated with each target bioactivity. Oyster antihypertensive peptides (AHPs) were selected based on AHTpin scores (>1.0) and predicted binding to all three active site residues of human angiotensin-converting enzyme (ACE1) with PepSite2; a full analysis is shown in Table 4. Candidate AHPs were further shortlisted based on predicted bioavailability indices, namely <30% predicted human intestinal absorption and <20% bioavailability score using ADMETlab2.0 (Supplementary Table S2).

Antidiabetic peptides (ADPs) were selected based on iDPPIV-SCM scores (> 350) with at least two binding sites for human dipeptidyl peptidase (DPPIV) when analyzed using PepSite2 (Table 5). High-confidence anti-inflammatory peptides (AIPs) were predicted by both PreAIP and Antiinflam were ranked to select the top four peptides. The top antimicrobial peptide (AMP) selection cutoffs were set at CAMP-SVM = 1, CAMP-RF > 0.54, ADAM > 2. The only positive hit in DBAASP v3′s General Antimicrobial Activity prediction result was also included. The three most recent and performance-optimized anti-cancer peptide prediction servers, ACPred, iDACP, and mACPpred were chosen from a wide range of available tools. Positive cross-hits from all three platforms were selected as the top three anti-cancer peptides.

## 3. Discussion

The ease and efficiency of in silico approach have fueled its increasing traction in the predictive screening of BAPs, especially combining with or preceding the traditional in vitro/in vivo experiments. Meanwhile, a growing and diversifying pool of in silico tools is being developed and improved. This presents a huge opportunity to define an adaptable workflow for effective BAP scouting using the latest tools. Pacific oyster has shown tremendous potential with their wide range of associated bioactivities [8]. Therefore, using *C. gigas* as a case study, we have outlined an applicable framework using existing publicly accessible in silico tools to efficiently scope for novel BAPs from commonly consumed seafood.

In silico proteolysis is an efficient way to simulate GI digestion, since the oyster proteins are often consumed directly. Both available platforms, BIOPEP Enzyme Action Tool and ExPASy PeptideCutter, present two sets of pepsin and chymotrypsin subtypes: pepsin at pH = 1.3 vs pH > 2; chymotrypsin A with low or high specificity. However, recent studies which adopted in silico simulated GI digestion used discordant enzyme choices without a clear explanation of the selections [3,25,26,27,28]. Such a variation presents a potential understanding gap in applying the platforms. In comparison, the human pepsin A3 information as documented in the peptidase database MEROPS (A01.001) better matches the cleavage preferences and lower specificity of pepsin at pH > 2 (Table 2). In addition, the lower specificity yields shorter and more digestion-tolerant peptide products, which is preferable for an overhaul of bio-accessible fragments. Similarly, human digestive chymotrypsin A’s catalytic activity from its MEROPS (S01.152) entry is a much better match to low specificity chymotrypsin A’s activity in the hydrolysis platforms and can produce highly digestive-tolerant peptides. Finally, the comparisons led to the best-aligned enzyme choices used for this study, indicated in Table 2. 

The peptide distribution post-digestion in Figure 1 shows muscle proteins, particularly myosin and paramyosin, yielded greater proportions of short peptides, which indicates higher digestibility with dietary relevance as muscle proteins are highly abundant in the flesh [15]. One caveat is that both digestion tools assume no protein folding which could restrict enzyme access. Under real-life gut conditions, there might be fewer cleavages made than virtually generated.

The outputs from both digestion tools, BIOPEP Enzyme Action Tool and ExPASy PeptideCutter were included collectively due to the observation that only a small portion of peptide results overlapped, with only di- and tripeptides, despite using the same set of enzymes. We suspected that the enzyme’s cleavage sites were encoded differently. As evident in Figure 1, BIOPEP generated predominantly di- and tripeptides, and very few oligopeptides. In fact, the sequence length range of five to ten residues has no overlapping results between the two platforms. Table 2 details the enzyme cleavage information cited in the respective server page, which shows slight variation between the two platforms both based on the references and the resulting sequences. Therefore, pooling all resulting peptides from both sources helps maximize the chance to identify potential bioactive peptides.

A peptide length range of 5 to 10 residues was determined to balance and maximize bioavailability and novelty. Bioactive peptides can transport from the gut lumen through the intestinal epithelium into the bloodstream, by four possible pathways: PepT1-mediated permeation, paracellular transport through tight junctions, transcytosis via vesicles, and passive transcellular diffusion [29]. Xu et al.’s review [29] also presented BAPs in this length range that can cross the Caco-2 cell monolayers via the above mechanisms, with high permeability coefficients comparable to shorter peptides. Another reason to exclude di-, tri-and tetra-peptides from our study is the ubiquity of short peptides which makes them more likely to be omnipresent in various food sources. For example, dipeptide VY possesses antihypertensive activity but is shared by sardines, seaweed, sesame, and royal jelly products among commercialized bioactive peptide products [2]. Therefore, our peptide size filter will help maximize novelty by uncovering new and uniquely oyster sequences.

From enzymatic digestion to preliminary screening against unwanted attributes, to final in silico bioactivity scoping, the peptide candidate pool has been narrowed down from 1800 total peptide products post-digestion, with 308 sequences in the 5 to 10 residue length range, to 151 unique non-toxic, non-bitter, stable, and novel peptide candidates, then finally to 22 top sequences with high potential bioactivities. Such a sequential funnel procedure can greatly improve the breadth and power of BAP screening. The selected activity prediction servers in this study utilize a variety of Machine Learning algorithm-based models. Top BAP candidates in the final shortlist have scored highly across multiple predictors, whose model construction and datasets have been vetted for reliability. In addition, PepSite2 analysis complements the predictions by modeling the extent of protein-peptide interaction for enzyme inhibitory mechanisms.

Amino acid and dipeptide compositions combined with physicochemical properties are also helpful to elucidate shared features and trends in new and known BAPs. The peptides with strong ACE-inhibitory activity likely contain C-terminal proline, hydrophobic, aliphatic, branched, aromatic, or positively charged residues, N-terminal branched chain, or hydrophobic residues [30,31,32,33]. In addition, proline-containing peptides generally help resist digestion [1,2]. The peptides with strong DPPIV-inhibitory activity likely contain F, R, and Y residues [34]. C-terminal proline and DPP-IV substrate motif at the N-terminal, Xaa-Pro, or Xaa-A may act as DPP-IV inhibitors [35,36]. Anti-inflammatory peptides benefit from hydrophobic residues at the termini, and high hydrophobic residue content has shown stronger NO inhibition ability in vitro [37,38]. They have also shown an association with positively charged residues, though inconsistent findings have been reported [39,40]. Antimicrobial peptides (AMPs) are generally amphiphilic with hydrophobic and cationic residues, mainly lysine and arginine, to facilitate interaction with the negatively charged microbial lipid cell membranes [41,42,43,44]. A subset of AMPs can also serve as anticancer peptides with selective membranolytic and cytolytic activities. These anticancer peptides generally contain 5–30 residues in length, and as with AMPs, hydrophobic and cationic (preferably by lysine) and amphiphilic properties are significant to their diverse modes of action [42,45,46], while cysteine-rich domains can form stabilized scaffolds to help maintain extracellular motifs and structures [46,47]. Our shortlisted peptides in Table 3 show general consistency with previously reported composition and property trends detailed above. Nevertheless, no conclusive causal relationship has been drawn between sequence-based propensities and bioactivities. Additional research investigating how the sequence, structure, and properties of BAPs enable their mechanistic action is warranted.

On the other hand, there remains an opportunity to strengthen the proposed framework by incorporating more in-depth 3-D structural analyses. The current study is limited to PepSite2 comparative assessment of the best docking model of protein-peptide interactions, which is applicable to only two out of five bioactivities assessed. Yuan et al.’s recent work on ACE- and DPP-IV-inhibitory peptides from *C. gigas* followed a similar investigation process but included a docking study analyzed based on CDOCKER interaction energy, a CHARMm-based molecular docking algorithm [48]. The CDOCKER scores indicate ligand binding affinities calculated at different poses of simulated receptor-ligand binding modes. The accuracy and reliability of such molecular docking methods in reproducing experimental observations have shown promise to elucidate molecular mechanisms of small ligands. Although we have narrowed down and identified different BAPs from the same source and digestion process, the in silico platform selection and parameter settings greatly influence the filtering results. For example, in contrast to Yuan et al.’s study, we opted not to use PeptideRanker, (http://bioware.ucd.ie/~compass/biowareweb/Serverpages/peptideranker.php/, accessed on 10 January 2022), a widely utilized general bioactivity predictor built on an N-1 Neural Network (N1-NN) model based on shared features across various functional classes of BAPs [49]. However, its three classes of BAPs only included antimicrobial peptides, peptide hormones, and peptide toxins/venom. The training dataset is made up of heavily AMPs, including sources from BIOPEP, PeptideDB, APD2, and CAMP. Therefore, within the five bioactivities of interest in this study, we expected PeptideRanker scoring to be skewed towards AMPs.

Future work following up this study may adopt and cross-check using optimized molecular docking software such as flexible CDOCKER and AutoDock [50,51,52], as well as quantitative structure-activity relationship (QSAR) analysis to complement the proposed screening strategy. For example, membrane permeability can be modeled for predicted AMPs with Gram-positive or Gram-negative bacteria, associated with probable antibacterial activity. Going forward, this framework shall be continually updated and optimized with the frequent release of new tools and findings. It may also branch out to include other production methods of BAPs such as via commercial enzymes and microbial fermentation. We also suggest future in vitro validation in two ways. From a top-down approach, enzyme digestion can be performed to mirror the in silico process in this study. Peptidomic analysis can be performed to compare the peptide product profile to that from BIOPEP Enzyme Action Tool and PeptideCutter results. Additionally, bioactivity assays can be performed on both hydrolysates and synthetic peptides of the top candidate sequences from this study to confirm predicted bioactivity and potency.

## 4. Materials and Methods

### 4.1. Protein Selection and Retrieval

*C. gigas* protein sequences and information were retrieved from the UniProt Knowledgebase (https://www.uniprot.org/uniprotkb/, accessed on 15 November, 2021). Only SwissProt-reviewed entries, and major proteins identified through proteomics from recent literature were included. From the SwissProt database, all nine manually annotated and reviewed protein sequences of *C. gigas* expressed in the oyster flesh were included. In addition to the database search, we also included the 5 prominent *C. gigas* proteins identified through SDS-PAGE and subsequent nanoLC-nanoESI-MS/MS analysis [15] to strengthen the abundant protein inclusion criteria. The information on all 13 proteins analyzed in this study is listed in Table 6.

### 4.2. Enzyme Hydrolysis Using BIOPEP and ExPASy PeptideCutter

Simulated human gastrointestinal (GI) digestion was performed with enzymes pepsin pH > 2 (EC 3.4.23.1), trypsin (EC 3.4.21.4), and chymotrypsin A (EC 3.4.21.1) in both programs, BIOPEP-UVM’s Enzyme Action Tool (https://biochemia.uwm.edu.pl/en/biopep-uwm-2/, accessed on 21 December 2021) and ExPASy PeptideCutter (https://web.expasy.org/peptide_cutter/, accessed on 21 December 2021). The unique peptide sequence outputs were collated. Peptides in the length range of 5 to 10 residues were included for further analysis.

### 4.3. Toxicity, Bitterness, Stability, and Allergenicity Screening

All virtually generated peptide products were subjected to multiple database searches as peptide queries in FASTA form. ToxinPred (https://webs.iiitd.edu.in/raghava/toxinpred/multiple_test.php/, accessed on 10 January 2022), a support vector machine (SVM)-based prediction method with a threshold score of 0.0, was chosen to screen for non-specific peptide cytotoxicity. Digestion peptide products predicted as toxic were excluded. iBitter-SCM (http://camt.pythonanywhere.com/iBitter-SCM/, accessed on 10 January 2022) was used as the bitterness screening tool. Any peptides scoring above the threshold of 333 were predicted to be bitter-tasting, and therefore excluded from subsequent analyses. PLifePred (https://webs.iiitd.edu.in/raghava/plifepred/, accessed on 1 February 2022) was used to assess peptide stability in blood. Peptides with predicted half-life values above 800 s are included. HLP (https://webs.iiitd.edu.in/raghava/hlp/, http://crdd.osdd.net/raghava/hlp/interactive.htm/, accessed on 1 February 2022) was used to assess peptide stability in the intestinal environment. Additionally, our peptide pool was checked against reported allergenic motifs of the major oyster allergen tropomyosin *Cra g1* [53,54,55], along with BIOPEP-UVM’s Allergenic Proteins and their Epitopes database, listed in Supplementary Table S1. No matches were found to be excluded from subsequent analysis. 

### 4.4. Bioactivity and Novelty Assessments

All non-toxic, non-bitter, stable, and nonallergenic oyster peptides were screened against bioactivity platforms and peptide repositories to favor the discovery of novel antihypertensive, antidiabetic, anti-inflammatory, antimicrobial, and anticancer peptides. Positive peptide hits for bioactivity that have not been recorded in the existing databases are extracted as the potential bioactive peptide longlist. The servers used are collated in Table 7.

These BAP candidates were then screened against BIOPEP-UVM, PepBank (http://pepbank.mgh.harvard.edu/, accessed on 10 March 2022), PeptideDB (http://www.peptides.be/, accessed on 10 March 2022), EROP-Moscow (http://erop.inbi.ras.ru/index.html/, accessed on 10 March 2022), and BioPepDB (http://bis.zju.edu.cn/biopepdbr/index.php/, accessed on 11 April 2022) to ensure that they are not yet recorded and reported.

In addition, PepSite2 (http://pepsite2.russelllab.org/, accessed on 5 May 2022) was adopted to predict the binding site interactions between BAP candidates and human ACE (PDB entry 1O86) or dipeptidyl peptidase-4 enzyme (DPP-IV) (PDB entry 2ONC) respectively, using Mohd Salim and Gan’s analysis procedure with minor modifications [17]. The top-ranked sequences and cross-hits between platforms for each bioactivity were finally selected as high-potential bioactive peptides, shortlisted in Table 3.

## 5. Conclusions

The present study presents an in silico-based approach to discover novel bioactive peptides from *C. gigas* by investigating a wide range of protein and peptide-level databases and tools. A promising bioactive peptide candidate shortlist is provided for antihypertensive, antidiabetic, anti-inflammatory, and anti-cancer activities. These novel BAPs are predicted to be non-toxic, non-bitter, bioavailable, and stable in the intestines and circulation. Based on their predictive bioactivities, it will be of value to validate these bioactivities and explore potency in terms of hydrolysate preparation.

## Figures and Tables

**Table 1:** Digestive enzyme selection in BIOPEP and ExPASy PeptideCutter.

Digestion Platform	Enzyme Name	EC Number	Cleavage Residues	Additional Information and References
BIOPEP Enzyme Action Tool	Pepsin (pH > 2)	3.4.23.1	C-terminus of F, L, G, Y, A, E, Q, T, N, K, D, M; N-terminus of V, I	MEROPS A01.001 show broad cleavage specificity
	Trypsin	3.4.21.4	C-terminus of K, R	P after K blocks enzyme action [20]
	Chymotrypsin (A)	3.4.21.1	C-terminus of F, Y, W, L, N, H, M	P after Y, P or M after W, P after F, P after L block enzyme action [21].
ExPASy PeptideCutter	Pepsin (pH > 2)	3.4.23.1	Broad specificity with preference at F, Y, W, L in position P1 or P1’ [20]	Lost specificity at pH >= 2
	Trypsin	3.4.21.4	Preferentially cleaves R and K at P1	P at P1′ blocks enzyme action but not when K and W or R and M are at P1 and P2 at the same time. If K at P1, enzyme action is blocked when D and D, C and D, C and H, or C and Y are at P2 and P1′ respectively. When R is at P1, enzyme action is blocked with R and H, C and K, or R and R are at P2 and P1′respectively.
	Chymotrypsin (low specificity)	3.4.21.1	Preferential at F, Y, W, L, M at P1	Exceptions: P at P1′; M or P at P1′ when is W is at P1; Y at P1′ when M is at P1; when H is at P1, D, M or W also blocks the cleavage.

**Table 2:** Peptide candidate long list pre-screened for size range, toxicity, bitterness, stability, and novelty.

Identifier				Toxicity		Bitterness		Stability			Novelty Finds			
Peptide No.	Source Protein Accession Number	Peptide Sequence	# Residues	ToxinPred		iBitter-SCM		PLifePred	HLP		BIOPEP Database	PepBank	EROP-MOSCOW	BioPepDB
FASTA				Score	Descriptor	Score	Descriptor	Half-Life in Blood (s)	Intestinal Half-Life (s)	Stability				
>O1	O17320	NSPAM	5	−0.58	Non-Toxin	308	non-Bitter	832.11	1.553	High	0	0	0	0
>O2	P0DUE1	PQSCR	5	−0.58	Non-Toxin	251.25	non-Bitter	826.31	1.425	High	0	0	0	0
>O3	A9XE49	ICPSS	5	−0.67	Non-Toxin	320.25	non-Bitter	833.81	1.358	High	0	0	0	0
>O4	XP_034310988.1	CTGAI	5	−0.63	Non-Toxin	285.25	non-Bitter	839.41	1.32	High	0	0	0	0
>O5	A9XE49	CEPVY	5	−0.86	Non-Toxin	229	non-Bitter	847.51	1.26	High	0	0	0	0
>O6	XP_034310988.1	QACID	5	−0.55	Non-Toxin	320.25	non-Bitter	840.71	1.477	High	0	0	0	0
>O7	P0DUE1	SVPVL	5	−0.97	Non-Toxin	283.25	non-Bitter	836.51	1.192	High	0	0	0	0
>O8	XP_034310988.1	QCNGV	5	−0.61	Non-Toxin	322.75	non-Bitter	826.41	1.513	High	0	0	0	0
>O9	XP_034310988.1	STHPH	5	−0.73	Non-Toxin	308	non-Bitter	834.81	1.292	High	0	0	0	0
>O10	XP_034310988.1	IEKPM	5	−0.82	Non-Toxin	330.25	non-Bitter	838.31	2.38	High	0	0	0	0
>O11	XP_034310988.1	AGSVP	5	−0.67	Non-Toxin	321.5	non-Bitter	913.01	1.316	High	0	0	0	0
>O12	Q4GWV4	CDAAT	5	−0.73	Non-Toxin	278	non-Bitter	835.01	1.239	High	0	0	0	0
>O13	Q4GWV4	VSADM	5	−0.8	Non-Toxin	327.5	non-Bitter	838.01	1.27	High	0	0	0	0
>O14	O17320	SSSSL	5	−0.8	Non-Toxin	312.75	non-Bitter	834.81	1.374	High	0	0	0	0
>O15	P0DUE1	SEPNI	5	−0.82	Non-Toxin	229	non-Bitter	832.51	1.595	High	0	0	0	0
>O16	O17320	TVPIY	5	−1.1	Non-Toxin	330.5	non-Bitter	823.91	1.402	High	0	0	0	0
>O17	P0DUE1	SPSST	5	−0.88	Non-Toxin	325.25	non-Bitter	835.01	1.249	High	0	0	0	0
>O18	XP_034310988.1	DAANR	5	−0.89	Non-Toxin	275.5	non-Bitter	835.61	1.251	High	0	0	0	0
>O19	Q20A06	CDAVT	5	−0.81	Non-Toxin	322.75	non-Bitter	831.71	1.054	High	0	0	0	0
>O20	XP_034310988.1	INQGA	5	−0.9	Non-Toxin	320	non-Bitter	845.01	1.68	High	0	0	0	0
>O21	XP_034310988.1	QSDVR	5	−0.72	Non-Toxin	318.5	non-Bitter	838.71	1.471	High	0	0	0	0
>O22	XP_011417566.1	IDQNR	5	−0.74	Non-Toxin	332.5	non-Bitter	858.61	1.386	High	0	0	0	0
>O23	XP_011417566.1	DKDGK	5	−0.71	Non-Toxin	325.25	non-Bitter	835.31	1.295	High	0	0	0	0
>O24	XP_034310988.1	SEIDR	5	−0.96	Non-Toxin	317.5	non-Bitter	824.71	1.375	High	0	0	0	0
>O25	XP_034310988.1	DNQIK	5	−0.82	Non-Toxin	302.75	non-Bitter	915.21	2.152	High	0	0	0	0
>O26	XP_034310988.1	AADER	5	−0.96	Non-Toxin	270.5	non-Bitter	865.31	1.306	High	0	0	0	0
>O27	XP_034310988.1	QQQIK	5	−0.89	Non-Toxin	308.25	non-Bitter	834.81	1.405	High	0	0	0	0
>O28	XP_034310988.1	ENQSM	5	−0.79	Non-Toxin	300.75	non-Bitter	840.31	1.834	High	0	0	0	0
>O29	XP_034310988.1	ANDNT	5	−0.78	Non-Toxin	276	non-Bitter	834.31	1.807	High	0	0	0	0
>O30	XP_034310988.1	GAVDK	5	−0.69	Non-Toxin	312.75	non-Bitter	832.31	1.239	High	0	0	0	0
>O31	Q95WY0	DEAAR	5	−0.99	Non-Toxin	278	non-Bitter	865.31	1.256	High	0	0	0	0
>O32	XP_011429256.1	DAAER	5	−0.95	Non-Toxin	268	non-Bitter	865.31	1.587	High	0	0	0	0
>O33	XP_034310988.1	DSEQR	5	−1	Non-Toxin	327.75	non-Bitter	846.21	2.5	High	0	0	0	0
>O34	XP_034310988.1	EGQIK	5	−0.77	Non-Toxin	269.25	non-Bitter	829.31	1.409	High	0	0	0	0
>O35	Q95WY0	DAENR	5	−0.7	Non-Toxin	332.75	non-Bitter	824.81	1.525	High	0	0	0	0
>O36	XP_034310988.1	VAANI	5	−0.96	Non-Toxin	256	non-Bitter	834.91	1.252	High	0	0	0	0
>O37	XP_011417566.1	AQQQK	5	−0.87	Non-Toxin	308.5	non-Bitter	834.81	1.405	High	0	0	0	0
>O38	XP_011429256.1	DDESR	5	−0.64	Non-Toxin	221.75	non-Bitter	834.31	1.567	High	0	0	0	0
>O39	XP_034310988.1	EQTQP	5	−1.18	Non-Toxin	273.25	non-Bitter	835.11	1.335	High	0	0	0	0
>O40	XP_011429256.1	SVSER	5	−1.02	Non-Toxin	300	non-Bitter	835.01	1.138	High	0	0	0	0
>O41	XP_011429256.1	ETDIR	5	−0.85	Non-Toxin	320	non-Bitter	836.41	1.07	High	0	0	0	0
>O42	XP_011429256.1	AAEER	5	−0.85	Non-Toxin	278	non-Bitter	839.41	2.234	High	0	0	0	0
>O43	XP_011429256.1	EANQA	5	−0.96	Non-Toxin	307.75	non-Bitter	852.71	1.884	High	0	0	0	0
>O44	XP_011429256.1	TEINR	5	−0.86	Non-Toxin	332.5	non-Bitter	830.21	1.509	High	0	0	0	0
>O45	XP_034310988.1	EQAER	5	−1.08	Non-Toxin	318	non-Bitter	818.61	2.042	High	0	0	0	0
>O46	P0DUE1	EESGK	5	−0.8	Non-Toxin	254.25	non-Bitter	832.51	1.8	High	0	0	0	0
>O47	XP_011429256.1	DAETK	5	−0.83	Non-Toxin	330	non-Bitter	834.81	1.67	High	0	0	0	0
>O48	XP_034310988.1	AEVTR	5	−0.71	Non-Toxin	330.25	non-Bitter	847.31	1.154	High	0	0	0	0
>O49	XP_011429256.1	VQVDD	5	−0.81	Non-Toxin	261.75	non-Bitter	834.81	1.804	High	0	0	0	0
>O50	Q95WY0	EETIR	5	−0.66	Non-Toxin	327.5	non-Bitter	832.91	1.541	High	0	0	0	0
>O51	XP_034310988.1	ITDEA	5	−0.94	Non-Toxin	332.5	non-Bitter	829.81	1.499	High	0	0	0	0
>O52	XP_034310988.1	AAESE	5	−0.89	Non-Toxin	197	non-Bitter	833.61	1.548	High	0	0	0	0
>O53	XP_034310988.1	EAEAK	5	−0.79	Non-Toxin	322.5	non-Bitter	835.71	1.455	High	0	0	0	0
>O54	XP_034310988.1	AETQK	5	−0.77	Non-Toxin	313	non-Bitter	856.11	1.544	High	0	0	0	0
>O55	Q95WY0	EEASK	5	−0.73	Non-Toxin	328.75	non-Bitter	831.11	1.462	High	0	0	0	0
>O56	XP_034310988.1	TESTK	5	−1.01	Non-Toxin	269	non-Bitter	817.31	2.175	High	0	0	0	0
>O57	A9XE49	CSGCVP	6	−0.72	Non-Toxin	305.8	non-Bitter	834.71	1.502	High	0	0	0	0
>O58	Q20A05	GCPGDQ	6	−0.64	Non-Toxin	327.2	non-Bitter	844.71	3.646	High	0	0	0	0
>O59	XP_034310988.1	SVTPSF	6	−0.95	Non-Toxin	312.4	non-Bitter	830.41	1.106	High	0	0	0	0
>O60	O17320	CDVDIR	6	−1.1	Non-Toxin	323	non-Bitter	828.71	1.446	High	0	0	0	0
>O61	XP_034310988.1	CIIPNE	6	−0.41	Non-Toxin	272.8	non-Bitter	834.21	1.451	High	0	0	0	0
>O62	XP_034310988.1	CVAINP	6	−0.63	Non-Toxin	272	non-Bitter	828.71	1.164	High	0	0	0	0
>O63	XP_034310988.1	DDIQQM	6	−1.08	Non-Toxin	259.6	non-Bitter	835.61	1.138	High	0	0	0	0
>O64	A9XE49	AAAVHM	6	−0.94	Non-Toxin	232	non-Bitter	834.91	1.269	High	0	0	0	0
>O65	XP_034310988.1	QQPAER	6	−1.37	Non-Toxin	276	non-Bitter	835.61	1.478	High	0	0	0	0
>O66	XP_011429256.1	ASADAK	6	−0.72	Non-Toxin	312	non-Bitter	834.91	1.164	High	0	0	0	0
>O67	O17320	ESSGIH	6	−0.87	Non-Toxin	274.6	non-Bitter	833.51	1.314	High	0	0	0	0
>O68	P0DUE1	ICNEIK	6	−0.7	Non-Toxin	329.2	non-Bitter	836.11	1.33	High	0	0	0	0
>O69	XP_034310988.1	QADEDR	6	−0.99	Non-Toxin	323.2	non-Bitter	839.91	1.874	High	0	0	0	0
>O70	XP_034310988.1	VSSSSM	6	−0.76	Non-Toxin	315	non-Bitter	834.91	1.376	High	0	0	0	0
>O71	XP_011429256.1	AAAQAA	6	−0.92	Non-Toxin	190.6	non-Bitter	834.81	1.337	High	0	0	0	0
>O72	XP_011429256.1	EEASGM	6	−0.94	Non-Toxin	320	non-Bitter	830.11	1.463	High	0	0	0	0
>O73	XP_034310988.1	ADQIDQ	6	−0.69	Non-Toxin	321	non-Bitter	835.91	1.355	High	0	0	0	0
>O74	Q95WY0	QECQTK	6	−0.54	Non-Toxin	304.6	non-Bitter	838.01	1.592	High	0	0	0	0
>O75	XP_011429256.1	ETAANM	6	−0.91	Non-Toxin	293.2	non-Bitter	835.71	1.302	High	0	0	0	0
>O76	XP_034310988.1	SQQIEK	6	−1.04	Non-Toxin	292.6	non-Bitter	848.01	2.262	High	0	0	0	0
>O77	XP_011429256.1	AAEVNR	6	−0.88	Non-Toxin	289.6	non-Bitter	852.51	1.335	High	0	0	0	0
>O78	P0DUE1	AVDDSH	6	−0.77	Non-Toxin	285.4	non-Bitter	832.71	2.198	High	0	0	0	0
>O79	XP_011429256.1	GETSQR	6	−1.06	Non-Toxin	300.4	non-Bitter	828.91	1.534	High	0	0	0	0
>O80	P0DUE1	DSVESR	6	−0.78	Non-Toxin	317.2	non-Bitter	834.11	2.269	High	0	0	0	0
>O81	XP_011417566.1	AATSNV	6	−0.98	Non-Toxin	255.2	non-Bitter	825.21	1.4	High	0	0	0	0
>O82	O17320	VAIQAV	6	−1.12	Non-Toxin	280.6	non-Bitter	835.01	1.432	High	0	0	0	0
>O83	XP_034310988.1	SAQVSS	6	−0.59	Non-Toxin	294.4	non-Bitter	842.51	1.323	High	0	0	0	0
>O84	XP_034310988.1	SQESTD	6	−0.95	Non-Toxin	257.6	non-Bitter	828.21	1.728	High	0	0	0	0
>O85	XP_034310988.1	IDECEE	6	−0.06	Non-Toxin	298	non-Bitter	834.81	1.455	High	0	0	0	0
>O86	XP_011429256.1	QAANES	6	−1.03	Non-Toxin	226.8	non-Bitter	836.41	1.3	High	0	0	0	0
>O87	Q95WY0	EAAEAK	6	−0.88	Non-Toxin	283.4	non-Bitter	837.01	1.426	High	0	0	0	0
>O88	XP_011429256.1	QVQVDD	6	−0.85	Non-Toxin	255.6	non-Bitter	834.81	1.518	High	0	0	0	0
>O89	Q95WY0	ATEAER	6	−1.16	Non-Toxin	319	non-Bitter	853.11	1.869	High	0	0	0	0
>O90	XP_034310988.1	EDAEER	6	−0.99	Non-Toxin	333	non-Bitter	832.01	1.855	High	0	0	0	0
>O91	XP_034310988.1	QAEEDK	6	−0.81	Non-Toxin	331.2	non-Bitter	847.91	2.09	High	0	0	0	0
>O92	Q95WY0	AITEVD	6	−1.17	Non-Toxin	301.4	non-Bitter	831.91	1.789	High	0	0	0	0
>O93	XP_034310988.1	EAQVSS	6	−0.89	Non-Toxin	300.4	non-Bitter	837.51	1.275	High	0	0	0	0
>O94	XP_034310988.1	ESENDE	6	−0.78	Non-Toxin	272.4	non-Bitter	835.11	1.929	High	0	0	0	0
>O95	Q95WY0	QTATEK	6	−1.13	Non-Toxin	306.4	non-Bitter	831.31	1.662	High	0	0	0	0
>O96	XP_011429256.1	VINTEK	6	−0.82	Non-Toxin	297.8	non-Bitter	850.91	1.557	High	0	0	0	0
>O97	XP_034310988.1	EEAEAA	6	−0.9	Non-Toxin	299.4	non-Bitter	834.91	1.561	High	0	0	0	0
>O98	XP_034310988.1	QTEQDS	6	−1.33	Non-Toxin	320.8	non-Bitter	834.11	2.184	High	0	0	0	0
>O99	O17320	TTTAER	6	−0.92	Non-Toxin	320.8	non-Bitter	834.71	1.464	High	0	0	0	0
>O100	P0DUE1	SPSSTNM	7	−0.83	Non-Toxin	328.5	non-Bitter	836.01	1.249	High	0	0	0	0
>O101	A9XE49	ICPSSIK	7	−0.8	Non-Toxin	313.67	non-Bitter	830.41	1.358	High	0	0	0	0
>O102	XP_034310988.1	DAEIANM	7	−0.56	Non-Toxin	320	non-Bitter	830.61	1.398	High	0	0	0	0
>O103	XP_011429256.1	EGDIAAM	7	−0.92	Non-Toxin	282	non-Bitter	846.91	1.856	High	0	0	0	0
>O104	XP_011417566.1	GSTPDDK	7	−0.57	Non-Toxin	304.5	non-Bitter	884.11	1.373	High	0	0	0	0
>O105	XP_034310988.1	ESDIQAM	7	−1.01	Non-Toxin	278.5	non-Bitter	875.81	1.265	High	0	0	0	0
>O106	XP_011429256.1	DSDIQTK	7	−1.39	Non-Toxin	315.33	non-Bitter	875.11	2.027	High	0	0	0	0
>O107	Q7M456	IPDSVVG	7	−1.04	Non-Toxin	314.33	non-Bitter	828.41	2.082	High	0	0	0	0
>O108	XP_034310988.1	AVADAAR	7	−0.94	Non-Toxin	284	non-Bitter	835.01	1.225	High	0	0	0	0
>O109	O17320	GDEDIAA	7	−0.88	Non-Toxin	283.67	non-Bitter	836.91	2.021	High	0	0	0	0
>O110	XP_034310988.1	SSVSVTR	7	−0.77	Non-Toxin	323.33	non-Bitter	835.21	1.211	High	0	0	0	0
>O111	P0DUE1	SDTPVTS	7	−1	Non-Toxin	290.33	non-Bitter	807.21	1.628	High	0	0	0	0
>O112	XP_034310988.1	ANTEVQM	7	−0.92	Non-Toxin	252	non-Bitter	829.71	1.641	High	0	0	0	0
>O113	XP_034310988.1	GDEITVK	7	−0.86	Non-Toxin	318.33	non-Bitter	833.81	2.4	High	0	0	0	0
>O114	XP_011417566.1	GSSSEEA	7	−0.49	Non-Toxin	318.33	non-Bitter	831.41	1.297	High	0	0	0	0
>O115	XP_034310988.1	TESIIAK	7	−0.73	Non-Toxin	296.33	non-Bitter	846.31	2.392	High	0	0	0	0
>O116	XP_011429256.1	ESTEASM	7	−0.88	Non-Toxin	283.5	non-Bitter	830.91	2.031	High	0	0	0	0
>O117	XP_034310988.1	EEAEAQA	7	−0.84	Non-Toxin	323.67	non-Bitter	837.51	1.561	High	0	0	0	0
>O118	XP_034310988.1	EEEQESK	7	−0.68	Non-Toxin	312.83	non-Bitter	834.91	1.716	High	0	0	0	0
>O119	XP_034310988.1	GPSSNPNF	8	−0.73	Non-Toxin	286.57	non-Bitter	832.41	1.282	High	0	0	0	0
>O120	P0DUE1	VDDLPPPL	8	−0.72	Non-Toxin	261.14	non-Bitter	835.51	2.204	High	0	0	0	0
>O121	XP_034310988.1	APNAIPQG	8	−0.61	Non-Toxin	280.71	non-Bitter	833.71	1.519	High	0	0	0	0
>O122	A9XE49	QEGCTCVR	8	−0.63	Non-Toxin	324.71	non-Bitter	806.11	1.643	High	0	0	0	0
>O123	Q7M456	YPPVHDNN	8	−0.59	Non-Toxin	296.57	non-Bitter	832.41	1.606	High	0	0	0	0
>O124	XP_034310988.1	QPGVIDAA	8	−1.31	Non-Toxin	318.14	non-Bitter	864.71	2.056	High	0	0	0	0
>O125	O17320	DSGDGVSH	8	−1.05	Non-Toxin	312.29	non-Bitter	823.31	3.856	High	0	0	0	0
>O126	O17320	VVDNGSGM	8	−0.57	Non-Toxin	326.71	non-Bitter	808.81	1.377	High	0	0	0	0
>O127	Q7M456	PSSDTESK	8	−0.89	Non-Toxin	290.29	non-Bitter	846.41	1.411	High	0	0	0	0
>O128	Q6L6Q6	QQGCNVNS	8	−0.45	Non-Toxin	306	non-Bitter	830.51	1.425	High	0	0	0	0
>O129	XP_034310988.1	IAGADIET	8	−0.65	Non-Toxin	320.86	non-Bitter	842.61	1.209	High	0	0	0	0
>O130	NP_001295835	SVVANNIK	8	−0.97	Non-Toxin	318.57	non-Bitter	818.21	1.147	High	0	0	0	0
>O131	XP_011429256.1	DEEIDSIR	8	−0.82	Non-Toxin	325	non-Bitter	833.01	1.544	High	0	0	0	0
>O132	XP_034310988.1	TSVSSSSM	8	−0.68	Non-Toxin	317.86	non-Bitter	835.51	1.094	High	0	0	0	0
>O133	XP_034310988.1	QTDTANEM	8	−0.54	Non-Toxin	324.57	non-Bitter	828.51	1.376	High	0	0	0	0
>O134	P0DUE1	DSIADESS	8	−0.71	Non-Toxin	283	non-Bitter	832.91	3.6	High	0	0	0	0
>O135	O17320	VGDEAQSK	8	−0.63	Non-Toxin	299.71	non-Bitter	902.11	2.04	High	0	0	0	0
>O136	XP_034310988.1	DEEDAAAD	8	−1.04	Non-Toxin	282.71	non-Bitter	835.01	1.586	High	0	0	0	0
>O137	O17320	TTAASSSS	8	−0.65	Non-Toxin	285.86	non-Bitter	834.91	1.159	High	0	0	0	0
>O138	Q4GWV4	CTCTDCNGK	9	−0.5	Non-Toxin	321	non-Bitter	819.11	1.392	High	0	0	0	0
>O139	O17320	VAPEEHPVL	9	−0.59	Non-Toxin	311.75	non-Bitter	828.01	2.088	High	0	0	0	0
>O140	XP_034310988.1	ASQDEVIAR	9	−1.14	Non-Toxin	309.12	non-Bitter	937.71	1.243	High	0	0	0	0
>O141	Q7M456	EVSETTCPR	9	−1.11	Non-Toxin	312.25	non-Bitter	800.31	1.3	High	0	0	0	0
>O142	XP_011429256.1	GTSPSTQNR	9	−1.05	Non-Toxin	330.25	non-Bitter	816.21	2.136	High	0	0	0	0
>O143	XP_034310988.1	ITGESGAGK	9	−1.28	Non-Toxin	278.5	non-Bitter	1058.51	1.721	High	0	0	0	0
>O144	XP_034310988.1	VGAEIQSSK	9	−0.92	Non-Toxin	308.25	non-Bitter	873.21	1.519	High	0	0	0	0
>O145	XP_034310988.1	EESQDSIEQ	9	−1.5	Non-Toxin	295.5	non-Bitter	838.81	1.576	High	0	0	0	0
>O146	XP_011429256.1	EEESESASN	9	−0.6	Non-Toxin	240.75	non-Bitter	834.81	1.517	High	0	0	0	0
>O147	XP_034310988.1	QTQIEEEQR	9	−1.08	Non-Toxin	326.25	non-Bitter	835.51	2.215	High	0	0	0	0
>O148	P0DUE1	ESSDDTTVCM	10	−0.2	Non-Toxin	303.67	non-Bitter	801.21	1.398	High	0	0	0	0
>O149	NP_001295835	QVQNDQASQR	10	−0.98	Non-Toxin	290.44	non-Bitter	838.51	2.22	High	0	0	0	0
>O150	XP_011429256.1	QIDEAEDVAN	10	−0.69	Non-Toxin	321.78	non-Bitter	877.01	1.482	High	0	0	0	0
>O151	XP_011429256.1	EIVTQAEDDR	10	−1.19	Non-Toxin	330.33	non-Bitter	852.41	1.067	High	0	0	0	0

**Table 3:** Top *C. gigas* bioactive peptide candidate shortlist with bioactivity prediction results.

Bioactivity	Identifier				Activity Prediction	
	Oyster Protein Source		Peptide Sequence	# Residues	Predictor	Score
	Accession Number	Name				
Antihypertensive	A9XE49	Interleukin 17-like protein (CgIL-17)	CEPVY	5	AHTpin	1.44
	XP_034310988.1	Myosin Heavy Chain, Striated Muscle	QQQIK	5		1.44
	O17320	Actin	VAPEEHPVL	9		1.37
	XP_011417566.1	Myosin Regulatory Light Chain B, Smooth Adductor Muscle Isoform X1	AQQQK	5		1.27
	XP_034310988.1	Myosin Heavy Chain, Striated Muscle	STHPH	5		1.02
DPPIV-Inhibitory	XP_034310988.1	Myosin Heavy Chain, Striated Muscle	EQTQP	5	iDPPIV-SCM	418.75
	Q7M456	Ribonuclease Oy (RNase Oy) (EC 3.1.27.-)	YPPVHDNN	8		373
	O17320	Actin	VAPEEHPVL	9		360
	O17320	Actin	NSPAM	5		359.25
	P0DUE1	Stimulator of interferon genes protein (TIR-STING) (Probable NAD(+) hydrolase) (EC 3.2.2.6)	SVPVL	5		350
Anti-inflammatory	Q4GWV4	Defensin Cg-Defm (Cg-Def) (Mantle defensin)	CTCTDCNGK	9	PreAIP	0.613
	XP_034310988.1	Myosin Heavy Chain, Striated Muscle	QACID	5	PreAIP	0.534
	A9XE49	Interleukin 17-like protein (CgIL-17)	QEGCTCVR	8	PreAIP	0.521
	P0DUE1	Stimulator of interferon genes protein (TIR-STING) (Probable NAD(+) hydrolase) (EC 3.2.2.6)	ICNEIK	6	PreAIP	0.491
					Antiinflam	3.356
Antimicrobial	XP_011429256.1	Paramyosin Isoform X2	EEESESASN	9	CAMP-SVM	1
					CAMP-RF	0.6545
					ADAM-SVM	-
	XP_011429256.1	Paramyosin Isoform X2	EEASGM	6	CAMP-SVM	1
					CAMP-RF	0.6395
					ADAM-SVM	-
	XP_011429256.1	Paramyosin Isoform X2	ETAANM	6	CAMP-SVM	1
					CAMP-RF	0.554
					ADAM-SVM	-
					ADAM-SVM	-
	XP_011417566.1	Myosin Regulatory Light Chain B, Smooth Adductor Muscle Isoform X1	DKDGK	5	CAMP-SVM	1
					CAMP-RF	0.5475
					ADAM-SVM	-
	Q95WY0	Tropomyosin (Allergen Cra g 1.03) (allergen Cra g 1) (Fragment)	SVVANNIK	8	DBAASPv3.0	-
					ADAM-SVM	-
Anti-cancer	A9XE49	Interleukin 17-like protein (CgIL-17)	CSGCVP	6	ACPred	0.947
					iDACP	0.5196
					mACPpred	0.9812
	Q20A06	Hemocyte defensin Cg-Defh1 (Fragment)	CQSIGCR	7	ACPred	0.935
					iDACP	0.5196
					mACPpred	0.8374
	Q4GWV4	Defensin Cg-Defm (Cg-Def) (Mantle defensin)	CTCTDCNGK	9	ACPred	0.985
					iDACP	0.5574
					mACPpred	0.9253

**Table 4:** PepSite2 analysis using ACE1 (PDB: 1O86) as the target protein.

Peptide Sequence	AHTpin SVM Score	Ranking/Selection Criteria	PepSite2 *p*-Value	Potential Binding Sites of ACE Protein	Potential Binding Residues on Peptide
CEPVY	1.44	AHTpin SVM Score > 1, Rank #1	6.94 × 10^−4^	H353, H383 *, H387 *, F409, H410, E411 *, A412, I413, G414, D415, F457, K511, H513, Y523, S526, F527, Q281, A354, E384, Y520, S355, Y146, F512, A356, Y146	C1, E2, P3, V4, Y5
QQQIK	1.44	AHTpin SVM Score > 1, Rank #2	1.37 × 10^−4^	Q281, H353, A354, H383 *	Q1, Q2, Q3, I4, K5
EVSETTCPR	1.43	AHTpin SVM Score > 1, Rank #3	6.96 × 10^−4^	Q281, H353, A354, S355, A356, H383 *, E384, H387 *, F391, E411 *, F457, K511, H513, Y520, H410, R522, W59, Y360	E1, V2, S3, E4, T5, T6, C7, P8, R9
VAPEEHPVL	1.37	AHTpin SVM Score > 1, Rank #4	2.30 × 10^−4^	Y146, W279, Q281, H353, H383 *, H387 *, H410, E411 *, A412, D415, F457, F460, K511, F512, H513, Y520, Y523, S526, F527, E384, A354, S355, A356, R522	V1, A2, P3, E4, E5, H6, P7, V8, L9
PQSCR	1.33	AHTpin SVM Score > 1, Rank #5	5.62 × 10^−5^	W279, Q281, H353, H383 *, E411 *, D415, F457, F460, K511, H513, Y520, Y523, S526, F527, A354, E384, H387 *, F409, H410, A412, I413, G414, Q530	P1, Q2, S3, C4, R5
AQQQK	1.27	AHTpin SVM Score > 1, Rank #6	7.32 × 10^−5^	Q281, H353, A354, H383 *, E384, H387 *, E411 *, F457, K511, H513, Y520, Y523, F527, W279, F460, F512	A1, Q2, Q3, Q4, K5
GPSSNPNF	1.17	AHTpin SVM Score > 1, Rank #7	1.07 × 10^−4^	W279, Q281, H353, A354, S355, H383 *, E384, H387 *, E411 *, F457, F460, K511, H513, Y520, Y523, N66, A356, W357, F391, F512, D358, Y360, F527	G1, P2, S3, S4, N5, P6, N7, F8
EQTQP	1.14	AHTpin SVM Score > 1, Rank #8	2.71 × 10^−5^	Q281, H353, A354, H383 *, E384, H387 *, E411 *, F457, F460, K511, H513, Y520, F527, F512, Y523, D415, S526, S355	E1, Q2, T3, Q4, P5
STHPH	1.02	AHTpin SVM Score > 1, Rank #9	5.55 × 10^−5^	Q281, H353, A354, S355, H383 *, E384, H387 *, E411 *, F457, H513, Y520, Y523, K511, W279, F460	S1, T2, H3, P4, H5

Notes: * denotes residue of active sites. The active site of ACE revolves around this zinc ion. Zinc ligands of ACE consist of H383, H387, E411 and a water molecule [22]. In addition, the water molecule was also bound to E384 via hydrogen bonds. These main stabilizing residues are Q281, H353, A354, K511, H513, Y520, and Y523 [22,23].

**Table 5:** PepSite2 analysis using DPP-IV (PDB: 2ONC) as the target protein.

Peptide Sequence	iDPPIV-SCM Score	Ranking/Selection Criteria	PepSite2 *p*-Value	Potential Binding Sites of DPP-IV (Enzyme Protein)	Potential Binding Residues (Peptide)
EQTQP	418.75	iDPPIV-SCM Score >= 350, Rank #1	5.18 × 10^−3^	Y48, W627, W629, V653, I703, I742, H748, I751, Y752, M755, Y547, S630 *, H740 *, G741	E1, Q2, T3, Q4, P5
TVPIY	389.25	iDPPIV-SCM Score >= 350, Rank #2	1.78 × 10^−2^	Y48, W627, W629, V653, I703, I742, H748, I751, Y752, M755, G741, F357, Y547, Y666	T1, V2, P3, I4, Y5
VDDLPPPL	385	iDPPIV-SCM Score >= 350, Rank #3	6.52 × 10^−2^	F357, Y547, P550, W629, Y631, Y666, Y670, G741, Y752, Y48, W627, H748	V1, D2, D3, L4, P5, P6, P7, L8
YPPVHDNN	373	iDPPIV-SCM Score >= 350, Rank #4	3.23 × 10^−2^	F357, Y547, P550, C551, Y585, W629, S630 *, Y631, Y662, Y666, Y670, H740 *, Y48, W627, H748, Y752, G741	Y1, P2, P3, V4, H5, D6, N7, N8
VAPEEHPVL	360	iDPPIV-SCM Score >= 350, Rank #5	8.93 × 10^−2^	F357, Y547, P550, W627, W629, S630 *, Y631, Y662, Y666, Y670, H740 *, Y752, Y48, M733, W734, Y735, H750, G741, H748	V1, A2, P3, E4, E5, H6, P7, V8, L9
NSPAM	359.25	iDPPIV-SCM Score >= 350, Rank #6	1.22 × 10^−2^	Y48, W627, W629, H740 *, Y752, H748, S630 *, Y631, V656, W659, Y662, Y666, Y547	N1, S2, P3, A4, M5
SVPVL	350	iDPPIV-SCM Score >= 350, Rank #7	8.85 × 10^−3^	Y48, W627, W629, H740 *, Y752, S630 *, G741, H748	S1, V2, P3, V4, L5

Notes: * denotes residue of active sites. The catalytic domain is from Q508 to P766 whereas the main catalytic residues are S630, D708, and H740 [24].

**Table 6:** Full list *C. gigas* proteins selection for analysis.

Database Source	Accession # ^a^	Protein Name	Length (Residues)	Mass (Da)
Swiss-Prot	Q4GWV4	Defensin Cg-Defm (Cg-Def) (Mantle defensin)	65	7008
	P0DUE1	Stimulator of interferon genes protein (TIR-STING) (Probable NAD(+) hydrolase) (EC 3.2.2.6)	415	47,031
	Q6L6Q6	Lysozyme (EC 3.2.1.17) (1,4-beta-N-acetylmuramidase) (Invertebrate-type lysozyme)	137	15,274
	Q20A05	Hemocyte defensin Cg-Defh2 (Fragment)	60	6439
	Q20A06	Hemocyte defensin Cg-Defh1 (Fragment)	60	6587
	Q7M456	Ribonuclease Oy (RNase Oy) (EC 3.1.27.-)	213	24,360
	Q95WY0	Tropomyosin (Allergen Cra g 1.03) (allergen Cra g 1) (Fragment)	233	26,867
	A9XE49	Interleukin 17-like protein (CgIL-17)	200	21,551
	O17320	Actin	376	41,792
NCBI RefSeq	XP_034310988.1	Myosin Heavy Chain, Striated Muscle	1986	222,660
	XP_011429256.1	Paramyosin Isoform X2	886	102,210
	NP_001295835	Tropomyosin Isoform X1	284	33,020
	XP_011417566.1	Myosin Regulatory Light Chain B, Smooth Adductor Muscle Isoform X1	166	20,580

^a^ Accession numbers were retrieved from UniProt Knowledgebase and NCBI Refseq databases respectively.

**Table 7:** Bioactivity prediction servers and databases used for bioactivity screening in the current study.

Platform Name	Purpose	URL	Citation
AHTpin	Anti-hypertensive peptide prediction	http://crdd.osdd.net/raghava/ahtpin/, accessed on 15 January 2022	[56]
iDPPIV-SCM	DPP-IV inhibitory peptide prediction	http://camt.pythonanywhere.com/iDPPIV-SCM/, accessed on 15 January 2022	[57]
PepSite2	Peptide-protein binding interaction modeling	http://pepsite2.russelllab.org/, accessed on 5 May 2022	[58]
PreAIP	Anti-inflammatory peptide prediction	http://kurata14.bio.kyutech.ac.jp/PreAIP/, accessed on 18 January 2022	[59]
AIPpred	Anti-inflammatory peptide prediction	http://www.thegleelab.org/AIPpred/, accessed on 1 March 2022	[60]
Antiinflam	Anti-inflammatory peptide prediction	http://metagenomics.iiserb.ac.in/antiinflam/, accessed on 1 March 2022	[61]
CAMP-R3	Antimicrobial peptide prediction	http://www.camp.bicnirrh.res.in/predict/, accessed on 20 January 2022	[62]
ACPred	Anti-cancer peptide prediction	http://codes.bio/acpred/, accessed on 11 March 2022	[63]
iDACP	Anti-cancer peptide prediction	http://mer.hc.mmh.org.tw/iDACP/, accessed on 11 March 2022	[64]
mACPpred	Anti-cancer peptide prediction	http://www.thegleelab.org/mACPpred/ACP.html, accessed on 13 March 2022	[65]
BIOPEP-UVM	Comprehensive BAP database	https://biochemia.uwm.edu.pl/en/biopep-uwm-2, accessed on 1 February 2022	[66]
PepBank	Comprehensive BAP database	http://pepbank.mgh.harvard.edu, accessed on 10 March 2022	[67]
PeptideDB	Comprehensive BAP database	http://www.peptides.be, accessed on 10 March 2022	[68]
EROP-Moscow	Comprehensive BAP database	http://erop.inbi.ras.ru/index.html, accessed on 10 March 2022	[69]
BioPepDB	Comprehensive BAP database	http://bis.zju.edu.cn/biopepdbr/index.php, accessed on 11 April 2022	[70]
AHTpDB	Anti-hypertensive peptide database	http://crdd.osdd.net/raghava/ahtpdb. accessed on 15 January 2022	[56]
BioDADPep	Anti-diabetic peptide database	https://omicsbase.com/BioDADPep. accessed on 11 April 2022	[71]
APD3	Antimicrobial peptide database	https://aps.unmc.edu, accessed on 20 January 2022	[72]
ADAM	Antimicrobial peptide database	http://bioinformatics.cs.ntou.edu.tw/adam, accessed on 12 May 2022	[73]
DBAASP v3.0	Antimicrobial peptide database	https://dbaasp.org, accessed on 12 May 2022	[74]
CancerPPD	Anti-cancer peptide database	http://crdd.osdd.net/raghava/cancerppd, accessed on 27 March 2022	[75]
TumorHoPe	Anti-cancer peptide database	https://webs.iiitd.edu.in/raghava/tumorhope/pepsearch.php, accessed on 27 March 2022	[76]

## Data Availability

Data are contained within the article.

## Supplementary Materials

The following supporting information can be downloaded at: https://www.mdpi.com/article/10.3390/molecules28020651/s1, Table S1: Allergenic motifs previously reported in Crassostrea gigas Tropomyosin Cra g 1. Table S2: Absorption parameters and physico-chemical properties of positively scored bioactive peptide candidates retrieved from ADMETlab2.0 and ProtParam.

## Sample Availability

Samples of the compounds are not available from the authors.

## Abbreviations

BAP	bioactive peptide
*C. gigas*	*Crassostrea gigas*
T2D	type 2 diabetes
DPP-IV	dipeptidyl peptidase-4
ACE	angiotensin-converting enzyme
SVM	support vector machine
RF	Random Forest
ADMET	absorption, distribution, metabolism and excretion properties, and toxicities
QSAR	quantitative structure-activity relationship
GI	gastrointestinal
