# Supplementary material for: In Silico Prospecting for Novel Bioactive Peptides from Seafoods: A Case Study on Pacific Oyster (Crassostrea gigas)
Source: Molecules. 2023 Jan 9;28(2):651. doi: 10.3390/molecules28020651 (PMC9867001; doi:10.3390/molecules28020651)
Supplement: Supplementary file 1 [file molecules-28-00651-s001.zip › molecules-2048452-supplementary.pdf]

In Silico **Prospecting for Novel Bioactive Peptides from Seafood: A Case Study on Pacific Oysters (*Crassostrea gigas*)**

Leyi Zhou[1], Rufa L. Mendez[1] and Jung Y. Kwon[1,2]

[1]Department of Food Science and Technology, College of Agricultural Sciences, Oregon State University, Corvallis, OR 97333, USA;

[2]Seafood Research and Education Center, Oregon State University, Astoria, OR 97103, USA

Address correspondence to JYK (Email: Jung.Kwon@oregonstate.edu)

Supplementary Table S1. Allergenic motifs previously reported in *Crassostrea gigas* Tropomyosin *Cra g* 1.

Cra g 1 **Motifs**	**Position**	**Citation**
TSLQKK	44-49	(Fang et al., 2019)
TKLEEAEKTASEAEQET	69-85	(Fang et al., 2019)
MERSEERLQT	99-108	(Fang et al., 2019)
NNASEERTDVL	134-144	(Fang et al., 2019)
VQNDQASQREDSYEET	209-224	(Fang et al., 2019)
IQLLEEDMERSEER	92-105	(Ishikawa et al., 1998)
DEERMDALENQLKEARMLAEEADRKYDEVARKLAMVE ADLERAEERAESG		(Marti et al., 2007)
IQLLEEDMERSEER	[41-54]	BIOPEP
LAITEVDLERAEARLEAAEAK	[118-138]	BIOPEP
SLEISEQEASQREDSYEETIRDLTQRLK	[155-182]	BIOPEP
LQKEVDRLEDEL	[198-209]	BIOPEP
KEVDRLEDE	[200-208]	BIOPEP
AISDELDQTFAEL	[218-230]	BIOPEP
AISDELDQTFAELAGY	[218-233]	BIOPEP

NRKVLENLNNASEERTDVLEKQL	[75-97]	BIOPEP
ENLNNASEERTDVLEKQLTEAKLIA	[80-104]	BIOPEP

Table S2. Absorption parameters and physico-chemical properties of positively scored bioactive peptide candidates retrieved from ADMETlab2.0 and ProtParam.

Peptide Sequence	Source Protein Accession #	Caco-2 Permeability (log scale)	HIA	F20%	F30%	Hydrophobicity	Hydropathicity	Hydrophilicity	Charge	Mol wt
EQTQP	Myosin Heavy Chain, Striated Muscle	-6.5	+++	++	+++	-0.45	-2.56	0.6	-1	601.68
YPPVHDNN	Ribonuclease Oy (RNase Oy) (EC 3.1.27.-)	-6.975	+	+++	+++	-0.25	-1.75	-0.11	-0.5	955.1
VAPEEHPVL	Actin	-6.974	+++	- -	+++	0.01	0.07	0.02	-1.5	990.25
NSPAM	Actin	-6.976	- -	++	+++	-0.09	-0.44	-0.26	0	518.64
SVPVL	Stimulator of interferon genes protein (TIR-STING) (Probable NAD(+) hydrolase) (EC 3.2.2.6)	-6.608	++	---	---	0.26	1.96	-0.9	0	513.7
CEPVY	Interleukin 17-like protein (CgIL-17)	-6.951	+	+++	+++	-0.02	0.06	-0.36	-1	609.75
QQQIK	Myosin Heavy Chain, Striated Muscle	-6.199	+	+++	+++	-0.49	-1.98	0.36	1	643.82
EVSETTCPR	Ribonuclease Oy (RNase Oy) (EC 3.1.27.-)	-7.594	+++	+++	+++	-0.35	-0.96	0.67	-1	1021.22
PQSCR	Stimulator of interferon genes protein (TIR-STING) (Probable NAD(+) hydrolase) (EC 3.2.2.6)	-7.169	+++	+++	+++	-0.55	-1.58	0.5	1	589.72
AQQQK	Myosin Regulatory Light Chain B, Smooth Adductor Muscle Isoform X1	-6.545	- -	+++	+++	-0.58	-2.52	0.62	1	601.73
GPSSNPNF	Myosin Heavy Chain, Striated Muscle	-7.209	++	+++	+++	-0.15	-1.17	-0.19	0	818.94
STHPH	Myosin Heavy Chain, Striated Muscle	-6.206	---	- -	+++	-0.26	-1.9	-0.22	1	577.66
CTCTDCNGK	Defensin Cg-Defm (Cg-Def) (Mantle defensin)	-8.017	++	+++	+++	-0.28	-0.58	0.27	0	944.17

QACID	Myosin Heavy Chain, Striated Muscle	-7.309	+	+++	+++	-0.08	0.36	-0.02	-1	548.67
QEGCTCVR	Interleukin 17-like protein (CgIL-17)	-7.238	++	+++	+++	-0.31	-0.43	0.29	0	895.12
ICNEIK	Stimulator of interferon genes protein (TIR-STING) (Probable NAD(+) hydrolase) (EC 3.2.2.6)	-7.488	+++	+++	+++	-0.14	0.1	0.27	0	718.95
CIIPNE	Myosin Heavy Chain, Striated Muscle	-7.263	+++	+++	+++	0.03	0.48	-0.23	-1	687.89
EEESESASN	Paramyosin Isoform X2	-7.951	+++	+++	+++	-0.41	-2.01	1.4	-4	981
EEASGM	Paramyosin Isoform X2	-7.325	++	+++	+++	-0.14	-0.75	0.75	-2	622.72
ETAANM	Paramyosin Isoform X2	-7.396	++	++	+++	-0.11	-0.37	0.08	-1	635.76
DKDGK	Myosin Regulatory Light Chain B, Smooth Adductor Muscle Isoform X1	-7.646	+++	+++	+++	-0.7	-3.04	2.4	0	561.65
SVVANNIK	Tropomyosin (Allergen Cra g 1.03) (allergen Cra g 1) (Fragment)	-7.236	+	+++	+++	-0.48	-1.98	0.92	0	616.74
CSGCVP	Interleukin 17-like protein (CgIL-17)	-7.587	+++	+++	+++	-0.2	-0.8	0.63	-2	753.86
CQSIGCR	Hemocyte defensin Cg-Defh1 (Fragment)	-6.5	+++	++	+++	-0.45	-2.56	0.6	-1	601.68

Notes: Optimal Caco-2 permeability > -5.15 log; (+) indicates Human Intestinal Absorption <30%, bioavailability <20% or <30% respectively. Color coding from red, yellow to green indicates increasing absorption rate and bioavailability.
